# Trust is a verb: how to reimagine confidence in health systems

**DOI:** 10.1093/heapro/daaf235

**Published:** 2026-01-13

**Authors:** Tina D Purnat, Elisabeth Wilhelm, Gloria Lihemo, David Scales

**Affiliations:** Harvard TH Chan School of Public Health, 677 Huntington Avenue, Boston, MA 02115, United States; Department of Midwifery, School of Healthcare and Care Sciences, University of West Attica, 28, Ag. Spyridonos str, Egaleo 12243, Greece; UNICEF Headquarters, 3 United Nations Plaza, New York, NY 10017, United States; Weill Cornell Medicine, 525 E 68th St, New York, NY 10065, United States

Trust in health, science, and public institutions has become a central concern in many countries (O’Doherty 2023, 2025 Edelman Trust Barometer 2025, Cologna *et al*. 2025, Larson and Bersoff 2025, Moran *et al*. 2025, Perlo and Cerise 2025, The Economist 2025, Younger-Khan 2025, Zhang *et al*. 2025). For decades, we treated trust like a bank account, like something institutions could deposit into, that it grows untouched and withdraw from at will. But that is not how trust works anymore, if it ever did.

Health and public health systems were designed to deliver care, monitor risks, and offer guidance that improves population wellbeing. But, people now move through shifting information environments that offer many more places to seek advice or affirmation (Eddy *et al*. 2014, Purnat *et al*. 2025). In the online spaces people learn from one another, form shared expectations, and build norms that feel responsive in ways top-down communication from institutions often does not. By the time they meet a clinician or encounter public health guidance, they have often spent considerable time researching and discussing health information.

Three shifts influence these expectations: (i) lived experience now guides how people make sense of health; (ii) conversations about science move at a pace that challenges understanding; and (iii) personalized tools and services shape what people view as credible. If health systems hope to build confidence in healthcare, prevention, and public health, they need to respond to these shifts. Trust is a verb. It is something we do in every interaction, not something we hold in reserve.

## Shift 1: Professional expertise must meet lived experience as a partner

People rarely arrive in clinical or community health settings without a story (Winters *et al*. 2025). They talk with family members, compare symptoms with friends, search online, and join support groups where people describe similar experiences. They follow influencers who offer recognition or practical advice. By the time they sit with a clinician or public health professional, they bring along an interpretation of their symptoms that reflects frustration, hope, and an effort to sense make their experience.

For example, women navigating menopause find online communities where influencers acknowledge their frustration with brain fog and exhaustion (Thomas *et al*. 2025). Commercial actors take advantage of this emotional landscape offering products that often lack evidence, yet resonate because the language mirrors lived experience (Wood *et al*. 2025).

Clinicians understand how much relational groundwork matters, but heavy workloads and short appointments have reduced the space and time to grow trust with careful listening and actions. When people cannot find that time or attention inside the health system, they look elsewhere. The resulting dynamic sits at the root of what many describe as medical gaslighting (Barnes 2023). Distrust does not come from misunderstanding science. Instead, it comes from feeling that your authority over your own experience has been dismissed. When someone says, ‘You're not listening,’ they're not rejecting evidence. They are asking for help making sense of what they already know. They want their lived experience to count. Trust grows when people and health professionals meet as partners in understanding rather than as competitors for truth.

## Shift 2: Conversation about health moves at a speed that strains understanding

People now learn about health in places where new information flows constantly, and the pace of that stream shapes what feels trustworthy. Scientific updates circulate beside fresher personal stories, political commentary, humor, opinion, and advertising. These pieces arrive in similar formats, which blur the distinctions that once helped people separate emerging ideas from established knowledge. At the same time, a person may learn more readily about a health topic from a short-form video feed than from a public health website. Influencers often present confident views in clips that feel personal and emotionally coherent, a contrast to slower, more formal updates from public health organizations, which use a tone that can feel distant from daily life.

This makes a difference when people try to make sense of scientific change. For example, during the pandemic, many watched guidance shift in real time. Each update reflected new evidence; yet, the shifts often felt chaotic. It resembled a patient watching clinical specialists debate treatment options, which can feel confusing if you do not understand the frame guiding discussion. Science now unfolds in public the same way. Without that frame, disagreement can look like chaos instead of progress.

## Shift 3: Personalization reshapes what feels credible

People now live in a time when almost everything adjusts to their preferences. Medicine promises treatments shaped by their genes (Stein 2025), while algorithms tailor content like music and search results to personal preferences (O'Gieblyn 2022, Muchmore 2025). This personalization feels empowering in an unpredictable world (Kümpel 2022). When so many parts of life bend to individual preferences, people begin expecting science to do the same. They look for health information that speaks to their own story, and the question shifts from ‘Is this true?’ to ‘Is this true for me?’

The want to control is often sold back to consumers. For example, home testing kits, wearable trackers, and personalized supplement plans promise agency and mastery over data, bodies, and decisions (McLean et al. 2025, Nickel *et al*. 2025). Yet, this sense of control also shapes what people believe science is for. As health information circulates through the same personalized streams as entertainment and opinion, people often encounter it as just another form of content shaped around their preferences rather than as knowledge produced within its own norms and purposes. Evidence expressed in averages feel impersonal, probabilities sound dismissive, and uncertainty starts to feel like a failure of care.

Here is the paradox: greater access to health information was supposed to empower people. Instead, it often places the entire burden of achieving wellness on individuals. Being unwell starts to feel like personal failure of choosing the wrong diet, missing a supplement, or making a choice you should have made differently. Empowerment becomes pressure (Singer 2026). In parallel, many health systems have promoted self-care and self-management as solutions, which can expand autonomy in some contexts but often transfer responsibility to individuals without providing the time, resources, or support needed to carry that burden well (Kitson and Lawless 2025).

This matters because it shapes how people interpret vulnerability. When health becomes a personal project, institutions built for collective wellbeing can seem irrelevant or even obstructive. In some settings, where quality is defined through population measures, people may feel that clinical targets or performance metrics leave little space for their own personal priorities, and experience this as pressure rather than a tailored solution. Rebuilding trust then means creating a clearer connection between the personal, (Bowen *et al*. 2025) shared values and the collective. The individual asks: ‘Is this true for me?’ Shared decision-making bridges that gap, connecting personal meaning to collective evidence. It answers: ‘Here is what is true for us.’ Trust grows in that space.

As these shifts shape how people interpret health information, they can also uncover where systems need to adapt. This brings us to the first pivot: how institutions act in ways that earn and sustain trust.

## Pivot 1: Practicing trustworthiness

Trust grows through what people see and experience over time. They pay close attention to how institutions act when situations feel uncertain, and they watch how health workers respond when someone feels overlooked or unsure. These encounters shape belief more than public statements because people judge systems through lived experience, especially when the stakes feel high and consequences are unevenly felt. Think of a nurse who takes an extra moment to explain why a treatment plan changed, or a public health official who says ‘we don't know yet’ instead of offering false certainty. These small acts matter more than polished communications campaigns.

Earning trust starts with how decisions take shape. It involves looking closely at how questions and disagreements are worked through, who has voice at different stages, and how responsibility is shared. In clinical practice, multidisciplinary reviews, ethics consultations, and team-based decisions involve diverse professionals that weigh uncertainty and examine tradeoffs, making reasoning explicit and visible (Bell *et al*. 2022).

This same principle extends to deliberative processes in public health. Countries have convened citizens to deliberate on topics such as vaccines, genomic screening, antimicrobial resistance, obesity policy, end-of-life decisions, newborn sequencing, universal health coverage, and drug-policy reform, and to discuss governance models, evaluation of policies and budgeting (Joss 1998, Henderson *et al*. 2013, Crowe *et al*. 2015, Victorian Health Promotion Foundation Melbourne 2016, Bispo and Serapioni 2021, France Assos Santé 2021, Participedia 2021, Scottish Government 2022, Katirai *et al*. 2023, Ford *et al*. 2024, Scottish Centre for Administrative Data Research 2025, Wagemakers *et al*. 2025). Participants listen to evidence, ask questions, and explore policy tradeoffs, within clearly defined roles and decision boundaries, taking the time to think and helping disagreement become constructive. When people are involved in ways that are meaningfully linked to decision making and understand how decisions form, they develop confidence in the process itself. Trust becomes visible when behavior matches values and when decisions reflect the principles institutions claim to uphold (Schirch 2023).

Institutions strengthen trust when they create the conditions that allow health workers to listen, explain, and respond with integrity, and when participatory practices are adequately resourced rather than symbolic. Trust in health professionals remains one of our strongest assets, yet it will remain only if we invest in them.

## Pivot 2: Renewing the social contract across generations

Trust feels different across generations because each group carries its own history of how they experienced health and authority. These experiences shape expectations about who should be trusted, how credibility is signaled, and what respectful engagement looks like. They also inhabit different information ecosystems through which they engage with health information (2025 Edelman Trust Barometer 2025).

Older adults often understand trust through steadiness, formality, and long relationships with clinicians. They grew up reading credibility through professional boundaries and visible signs of competence. For many, reliability and institutional consistency remain central to confidence in care.

Gen Xers grew up at a time when institutions overlooked concerns about women’s health, HIV, and environmental risks. That history bred skepticism. They expect transparency, empathy, and acknowledgment of harm.

Millennials and Gen Z learned to navigate information the way earlier generations learned to navigate libraries by comparing sources, cross-checking claims, and trusting people over institutions. For them, a responsive DM from a clinician can feel as meaningful as an appointment.

Renewing the social contract begins with recognizing these generational differences, listening across them, and working intergenerationally. It underscores the importance of building social capital across different ages as a foundation for trust building (The Trust for Civic Life 2025). What can appear as resistance or disengagement is often a request for dialogue grounded in respect and recognition.

## Pivot 3: Rebuilding the structures that connect science and society

Public health depends on people understanding how evidence becomes guidance. We have lost the structure that made that visible. For much of the twentieth century, scientific debates unfolded in bounded settings like journals and conferences, with the public encountering outcomes rather than the provisional judgments behind them.

Today, those boundaries have largely disappeared (Marwick and boyd 2011) as communication has shifted from factual accuracy to social relevance. Scientists, policymakers, journalists, influencers, and the publics now respond to emerging studies in the same online spaces. As context collapses, the distinctions between scientific debate, policy deliberation, and public discussion flatten as well. Cues that once signaled what kind of conversation was taking place are lost. Scientific expertise, clinical judgment, lived experience, and personal interpretation appear together with little guidance about which form of knowledge fits which task. If trust is to grow, people need clearer pathways that show how evidence moves from discovery to decision to public dialogue, and where uncertainty, judgment, and values enter along the way (Eddy *et al*. 2014).

Clinical research offers one example of how evidence generation has opened up to patients while retaining structure for discussion. When patients and advocates help shape study design, trials often recruit and retain participants more effectively and measure outcomes that matter in daily life. At the same time, scientists ensure the research questions, methods, and interpretation remain sound. Collaboration works best when roles are understood. This adds respect to good methodology. It says: your experience shapes the questions worth asking, and scientific rigor determines how to answer them reliably.

Comparable frames are needed beyond research, particularly in the spaces that link scientific dialogue with public conversation without collapsing the two. Science media centers in some countries already help journalists and the public understand new findings before they spread widely (Science Media Centre 2025). They add context, clarify uncertainty, and signal when debates are provisional rather than settled. Even so, media infrastructure cannot sustain this connective function alone. Communicators, trusted intermediaries, and community leaders also play a critical role by translating evidence into contexts that feel relevant, while preserving the integrity of scientific reasoning and situating it within everyday concerns and values.

The largest gap lies between scientific debate and public policy, where evidence is balanced against practicality, equity, and feasibility; yet, the process remains largely invisible. Strengthening this translational space would allow scientists, policymakers, practitioners, and people with lived experience to examine evidence together and identify the tradeoffs that shape real policy choices. This work depends not only on participation but also on clarity of purpose, defined roles, and visible reasoning, so that involvement meaningfully shapes decisions rather than merely accompanies them. Building on deliberative and participatory approaches such as citizen juries, community advisory boards, and community dialogues, shared reasoning could become a routine part of how guidance and policy are formed.

Rebuilding these structures restores the context that gives conversations shape and intention. Clearer boundaries between discovery, policy formation, and public dialogue allow discussions to unfold at a pace and depth suited to their purpose. Disagreement becomes easier to interpret and less likely to be mistaken for disorder. These boundaries also make room for more diverse participants, who can engage with greater confidence when they understand the nature of the discussion and the kind of contribution being invited. As people can follow how choices take form, they gain firmer ground for trust.

## Trust is a verb

Rebuilding confidence in health and science depends on how we show up in the everyday work of care, communication, and decision-making. Trust lives in action. It grows when professionals explain decisions, acknowledge uncertainty, and listen to the meanings people bring with them. It weakens when systems stretch workers thin or hide the reasoning behind choices.

Confidence is built through small, steady acts of clarity and fairness. Acting in ways worthy of trust, listening across generations, and strengthening the spaces where people reason together are all forms of this work. These practices build relationships that match the realities of how people now understand evidence and health.

Trust is a verb. It lives in the doctor who says ‘Let's figure this out together,’ and in the public health official who shows their reasoning instead of just their conclusions. It lives in every moment we choose clarity over convenience, partnership over prescription.

These are daily practices of small, steady acts that rebuild confidence one interaction at a time. When institutions commit to these habits and when they invest in the people doing this work, they create the systems and conditions for trust to take root and grow.

## Data Availability

Not applicable.
